# Supplementary material for: Genome-wide identification, characterization and gene expression of BES1 transcription factor family in grapevine (Vitis vinifera L.)
Source: Sci Rep. 2023 Jan 5;13:240. doi: 10.1038/s41598-022-24407-y (PMC9816167; doi:10.1038/s41598-022-24407-y)
Supplement: Supplementary file 3 — Supplementary Information. [file 41598_2022_24407_MOESM3_ESM.zip › Vvi_Atr/Vitis_vinifera.PN40024.v4.dna_sm.toplevel.fa.vs.Amborella_trichopoda.AMTR1.0.dna_sm.toplevel.fa.html/Atr-AmTr_v1.0_scaffold00160.html]

|  |  |  |  |  |  |  |  |  |  |  |  |  |  |
| --- | --- | --- | --- | --- | --- | --- | --- | --- | --- | --- | --- | --- | --- |
| Duplication depth | Reference chromosome | Collinear blocks | | | | | | | | | | | |
| 0 | Atr-ERN10968 |  |  |  |  |  |  |  |  |  |  |  |  |
| 0 | Atr-ERN10969 |  |  |  |  |  |  |  |  |  |  |  |  |
| 0 | Atr-ERN10970 |  |  |  |  |  |  |  |  |  |  |  |  |
| 0 | Atr-ERN10971 |  |  |  |  |  |  |  |  |  |  |  |  |
| 0 | Atr-ERN10972 |  |  |  |  |  |  |  |  |  |  |  |  |
| 0 | Atr-ERN10973 |  |  |  |  |  |  |  |  |  |  |  |  |
| 0 | Atr-ERN10974 |  |  |  |  |  |  |  |  |  |  |  |  |
| 0 | Atr-ERN10975 |  |  |  |  |  |  |  |  |  |  |  |  |
| 0 | Atr-ERN10976 |  |  |  |  |  |  |  |  |  |  |  |  |
| 0 | Atr-ERN10977 |  |  |  |  |  |  |  |  |  |  |  |  |
| 0 | Atr-ERN10978 |  |  |  |  |  |  |  |  |  |  |  |  |
| 0 | Atr-ERN10979 |  |  |  |  |  |  |  |  |  |  |  |  |
| 0 | Atr-ERN10980 |  |  |  |  |  |  |  |  |  |  |  |  |
| 0 | Atr-ERN10981 |  |  |  |  |  |  |  |  |  |  |  |  |
| 0 | Atr-ERN10982 |  |  |  |  |  |  |  |  |  |  |  |  |
| 0 | Atr-ERN10983 |  |  |  |  |  |  |  |  |  |  |  |  |
| 0 | Atr-ERN10984 |  |  |  |  |  |  |  |  |  |  |  |  |
| 0 | Atr-ERN10985 |  |  |  |  |  |  |  |  |  |  |  |  |
| 0 | Atr-ERN10986 |  |  |  |  |  |  |  |  |  |  |  |  |
| 0 | Atr-ERN10987 |  |  |  |  |  |  |  |  |  |  |  |  |
| 0 | Atr-ERN10988 |  |  |  |  |  |  |  |  |  |  |  |  |
| 0 | Atr-ERN10989 |  |  |  |  |  |  |  |  |  |  |  |  |
| 0 | Atr-ERN10990 |  |  |  |  |  |  |  |  |  |  |  |  |
| 0 | Atr-ERN10991 |  |  |  |  |  |  |  |  |  |  |  |  |
| 0 | Atr-ERN10992 |  |  |  |  |  |  |  |  |  |  |  |  |
| 0 | Atr-ERN10993 |  |  |  |  |  |  |  |  |  |  |  |  |
| 0 | Atr-ERN10994 |  |  |  |  |  |  |  |  |  |  |  |  |
| 0 | Atr-ERN10995 |  |  |  |  |  |  |  |  |  |  |  |  |
| 0 | Atr-ERN10996 |  |  |  |  |  |  |  |  |  |  |  |  |
| 0 | Atr-ERN10997 |  |  |  |  |  |  |  |  |  |  |  |  |
| 0 | Atr-ERN10998 |  |  |  |  |  |  |  |  |  |  |  |  |
| 0 | Atr-ERN10999 |  |  |  |  |  |  |  |  |  |  |  |  |
